# The profile of soluble anti-antigen antibodies detected by Immundot: Experience of the Immunology laboratory, University Hospital Hassan II-Fez, Morocco

**DOI:** 10.5339/qmj.2023.sqac.29

**Published:** 2023-11-26

**Authors:** Kaaouch Hanae, Ouboks Mohamed, El Mitri Ibrahim, Bhallil Ouahiba

**Affiliations:** ^1^Immunology Department, Central Medical Analysis Laboratory, University Hospital Hassan II, Fez, Morocco Email: hanae.kaaouch@gmail.com; ^2^Faculty of Medicine, Dentistry and Pharmacy, Sidi Mohamed Ben Abdellah University, Fez, Morocco

## Abstract

**Introduction:** Soluble anti-antigen antibodies (Anti-ENA) represent a specificity of anti-nuclear autoantibodies (ANA), which are directed against nuclear particles composed of small RNA (Ribonucleic acid) and proteins. They are found during certain autoimmune diseases (ADs), most frequently during systemic lupus erythematosus (SLE) and Gougerot syndrome. The aim of this study is to retrospectively analyze the positive serum for Anti-ENA antibodies and associated pathologies.

**Materials and methods:** This study was carried out at the Immunology Department, Central Laboratory of Medical Analysis, University Hospital Hassan II - Fez, Morocco, for a period of 2 years. 821 serum samples were included. The indirect immunofluorescence method (IFI) was used to search the ANAs in the serums. In the case of a positive result, an identification of the antigenic targets was performed by the Immunodot technique according to the supplier’s recommendations (D-Tek).

**Results:** Eighty-eight serums were found positive for anti-ENA antibodies. The mean age in our series was 41 ±15.5 years. There were 80 women and 8 men with a sex ratio F/M of 10. The results by associated pathologies show a strong association of the anti-ENA antibodies’ positivity with lupus and Gougerot syndrome.

**Conclusion:** Anti-ENA antibodies are important immunological markers for lupus and Gougerot syndrome diagnosis.

## References

[1] Charki Chakar (2019). Le profil des anticorps antinucleaires dans les maladies auto-immunes systemiques. Universite Mohammed V-Rabat Faculte de Medecine et de Pharmacie RABAT.

[2] Theander E (2015;). Prediction of Sjogren’s Syndrome Years Before Diagnosis and Identification of Patients With Early Onset and Severe Disease Course by Autoantibody Profiling. Arthritis Rheumatol.

